# Circulating Autoantibodies in Adults with Hashimoto’s Thyroiditis: New Insights from a Single-Center, Cross-Sectional Study

**DOI:** 10.3390/diagnostics14212450

**Published:** 2024-10-31

**Authors:** Omar Tripolino, Maria Mirabelli, Roberta Misiti, Antonio Torchia, Denise Casella, Francesco Dragone, Eusebio Chiefari, Marta Greco, Antonio Brunetti, Daniela P. Foti

**Affiliations:** 1 Department of Experimental and Clinical Medicine, University “Magna Græcia” of Catanzaro, 88100 Catanzaro, Italy; 2 Department of Health Sciences, University “Magna Græcia” of Catanzaro, 88100 Catanzaro, Italy; 3 Operative Unit of Clinical Pathology, “Renato Dulbecco” Hospital, 88100 Catanzaro, Italy

**Keywords:** Hashimoto’s thyroiditis, autoantibodies, cytokines, inflammation

## Abstract

Background: Hashimoto’s thyroiditis (HT) is a common autoimmune thyroid disorder characterized by elevated anti-thyroid peroxidase (A-TPO) antibodies. HT frequently coexists with other autoimmune conditions, which are marked by organ-specific and non-organ-specific autoantibodies, reflecting a deregulated immune response. However, the burden and clinical significance of these circulating autoantibodies in adult patients with HT remains unclear. Methods: A cross-sectional study was conducted at the University Hospital “R. Dulbecco” in Catanzaro, Italy, from November 2023 to May 2024, involving 200 euthyroid adults. The study population comprised 100 A-TPO-positive HT patients and 100 A-TPO-negative controls, matched for age and sex. Laboratory assessments included thyroid function tests and detection of autoantibodies [e.g., antinuclear antibodies (ANA), anti-parietal cell antibodies (APCA), and anti-neutrophil cytoplasmic antibodies (ANCA)]. Cytokine profiles were also measured using sensitive chemiluminescent multi-array technology. Results: HT patients were predominantly female (77.0%) with a median age of 56 years. Compared to controls, HT patients had higher median thyroid stimulating hormone (TSH) levels (2.215 vs. 1.705 μIU/mL, *p* = 0.025). Circulating autoantibodies were more prevalent in the HT group, with higher rates of APCA positivity (16.3% vs. 4.1%, *p* = 0.008) and atypical ANCA positivity (27.3% vs. 10.2%, *p* = 0.003). This suggests an increased risk for autoimmune gastritis and systemic inflammation. Additionally, HT patients with positive atypical ANCA showed elevated inflammatory cytokines, particularly interleukin-1 alpha (IL-1α), in female patients (*p* = 0.035). Conclusions: HT is significantly associated with a higher prevalence of circulating autoantibodies, such as APCA and atypical ANCA, which may indicate a heightened risk for autoimmune gastritis and broader autoimmune involvement. Detecting these autoantibodies in HT patients could serve as markers for more severe autoimmune dysfunction. These findings emphasize the need for proactive screening, especially in older patients and those with elevated A-TPO levels. Further research is essential to better understand the clinical implications and develop targeted management strategies for these patients.

## 1. Introduction

Autoimmune thyroid diseases (AITD) include two main clinical entities, Graves’ disease (GD) and Hashimoto’s thyroiditis (HT) [1]. HT is the most common autoimmune disease, affecting 7.5% of the general population [2]. It is the leading cause of hypothyroidism in adults, occurring in approximately 20–30% of affected individuals [3]. HT is more prevalent among Caucasians and Asians compared to African Americans, with females predominantly affected, particularly in middle-age [3,4].

From a pathological point of view, autoimmune diseases are characterized by a loss of self-tolerance due to an imbalance between regulatory and effector T and B cells. Th1 and Th17 cells, along with certain cytokines, play detrimental roles in many autoimmune conditions, including AITD [5,6,7,8,9,10]. In HT, a direct T-cell attack on thyroid tissue leads to thyroiditis and exposure to thyroid antigens, particularly thyroid peroxidase and thyroglobulin, against which autoantibodies are subsequently produced [5]. HT is therefore marked by the presence of anti-thyroid peroxidase (A-TPO) and anti-thyroglobulin (A-Tg) antibodies. Circulating A-TPO is detected in about 90% of HT patients and in approximately 50–60% of those with GD, providing 90% sensitivity for diagnosing AITD. In contrast, A-Tg is positive in 60–80% of HT patients, with a lower sensitivity (30–50%) and less specificity than A-TPO [3]. Unlike A-Tg, A-TPO antibodies are cytotoxic and may contribute to the immune pathogenesis of hypothyroidism [3,11].

The autoimmunity in HT is driven primarily by genetic, endogenous, and environmental factors, with genetics being the most influential [5,8]. Gene polymorphisms in major histocompatibility genes (HLA class I and II) and immunoregulatory genes (*CTLA-4*, *PTPN22*, *IL2RA*) have been implicated [1,8]. The strong genetic influence may explain why immune system dysregulation, marked by specific cell clusters and cytokines, is common across multiple autoimmune diseases, which often co-occur in the same individual. This concept is supported by epidemiological studies [12,13], and terms like “polyautoimmunity” [14] and “multiple autoimmune syndrome” [15] emphasize both the coexistence and potential common etiology of these conditions.

Patients with HT are at five times greater risk for developing another autoimmune disorder, either systemic or organ-specific. The exact prevalence of concomitant diseases varies depending on the population studied, study design, and detection methods [5,12,13]. In a cross-sectional study, Boalert et al. reported that 14.3% of HT patients had another autoimmune disorder, with rheumatoid arthritis being the most common coexisting condition. Relative risks for other autoimmune diseases, such as pernicious anemia, systemic lupus erythematosus (SLE), Addison’s disease, celiac disease, and vitiligo, were also significantly elevated [16]. Interestingly, the co-occurrence of HT with other autoimmune disorders differs between childhood and adulthood [17]. Additionally, HT can develop as part of autoimmune polyendocrine syndromes which are often diagnosed late [18,19].

In general, the association between autoimmune disorders may be underrecognized by clinicians, affecting prognosis, and potentially leading to poorer treatment outcomes [20,21,22]. However, autoimmune seropositivity does not always equate to disease development, as factors like inflammation, lymphoproliferative disorders, or certain therapies can induce autoantibody production, which can lead to misinterpretation [23]. Although clinical guidelines are lacking, these considerations underscore the need for an improved understanding of autoimmune comorbidities linked to HT.

This cross-sectional study aims to evaluate a panel of circulating autoantibodies in A-TPO-positive patients from a local Caucasian adult population in Calabria, Southern Italy. Our goal is to provide new insights into the significance and association of concomitant autoantibody occurrence in patients with HT.

## 2. Materials and Methods

### 2.1. Study Design and Population Selection

This cross-sectional study was conducted at the University Hospital “R. Dulbecco” in Catanzaro, Italy, from November 2023 to May 2024. We enrolled 200 euthyroid adults (aged over 18 years) from the local population who required simultaneous measurements of TSH, thyroid hormone levels, A-TPO, and A-Tg antibodies, with blood counts performed as needed. Euthyroidism was defined as normal thyroid hormone levels with a TSH range of 0.55–4.78 mIU/L. The case group comprised patients who tested positive for A-TPO, with or without A-Tg, while the control group included 100 matched individuals who were negative for both A-TPO and A-Tg. Exclusion criteria included patients who were positive only for A-Tg (but negative for A-TPO) or those attending the hospital for infectious or oncological conditions. The primary outcome was to assess the prevalence of autoimmune comorbidities based on circulating autoantibodies in patients with and without HT. Secondary outcomes included evaluating the clinical significance of autoantibody positivity in the context of HT.

### 2.2. Laboratory Assessments

Participants underwent fasting venipuncture in the morning for whole blood collection. The serum was prepared by centrifuging clotted blood at 3500× *g* for 10 min, then immediately aliquoted to assess thyroid function. This assessment included TSH, FT3, FT4, A-TPO, and A-Tg antibodies, using chemiluminescent immunoassays on the ADVIA Centaur^®^ system (Siemens Healthcare Diagnostics Inc., Camberley, UK) [24]. Negative cut-off values for A-Tg and A-TPO were established at <4.5 IU/mL and <60 IU/mL, respectively, based on the manufacturer’s specifications. Additional serum aliquots were stored at −80 °C for further autoimmune and cytokine testing. A-TSH-Receptor (A-TSH-R) antibodies were searched using the Immulite 2000 system, which employs a chemiluminescent immunoassay specific for thyroid-stimulating immunoglobulins. Blood counts were performed using the ADVIA 2120i system (Siemens Healthcare Diagnostics Inc., Camberley, UK). Autoimmune parameters and cytokines were measured in thawed serum samples during a few grouped analytical sessions to minimize variability. Anti-parietal cell antibodies (APCA), antinuclear antibodies (ANA), antineutrophil cytoplasmic antibodies (ANCA), anti-mitochondrial antibodies (AMA), and anti-adrenal cortex antibodies (ADA) were detected via indirect immunofluorescence assays (IIF) using Euroimmun commercial Kits (Euroimmun Medizinische Labordiagnostika AG, Lübeck, Germany). The Connective Tissue Disease Screen (EliA™ CTD Screen, ThermoFisher Scientific, Waltham, MA, USA), which tests for antibodies against U1RNP (RNP70, A, C), SS-A/Ro (60 kDa, 52 kDa), SS-B/La, Centromere B, Scl-70, Jo-1, Fibrillarin, RNA Pol III, Rib-P, PM-Scl, PCNA, Mi-2, Sm, and DNA, was interpreted according to the cut-off values specified by the manufacturer (>1.0 ratio, positive; 0.7–1.0 ratio, equivocal; <0.7 ratio, negative). Additional tests included anticyclic citrullinated peptide (ACCP), anti-tissue transglutaminase IgA/IgG, anti-Saccharomyces cerevisiae IgA/IgG (ASCA, ASCG), and anti-intrinsic factor antibodies (IFAB), performed using fluorimetric enzyme-linked immunoassays (FEIA) with ThermoFisher commercial kits on Phadia 250 (ThermoFisher Scientific, Waltham, MA, USA). Diabetes-related antibodies (GAD, IA2, ZNT8) were measured using enzyme-linked immunosorbent assays (ELISA) with Euroimmun commercial kits (Euroimmun Medizinische Labordiagnostika AG, Lübeck, Germany). Simultaneous quantification of serum cytokines and growth factors, including interleukin (IL)-1 alpha, IL-1 beta, IL-2, IL-4, IL-6, IL-8, IL-10, monocyte chemoattractant-1 (MCP-1), interferon (IFN)-gamma, tumor necrosis factor (TNF)-alpha, epidermal growth factor (EGF), and vascular endothelial growth factor (VEGF), was performed using the Randox preconfigured chemiluminescent immunoassay “Cytokine array I” kit and the Evidence Investigator biochip analyzer (Randox Laboratories Ltd., Crumlin, UK) [25]. All samples were processed centrally at the Clinical Pathology Laboratory, University Hospital of Catanzaro. For the autoimmune screening and cytokine assays, the intra-assay and inter-assay coefficients of variation (CV) were <5% and <10%, respectively.

### 2.3. Statistical Analysis

Demographic and laboratory data of the participants were presented either as the median with interquartile range or as counts with percentages (valid percentages were provided for variables with missing data in specific groups). Statistical comparisons between patients with positive and negative autoantibody statuses (including A-TPO, APCA, and ANCA) were performed using the Mann–Whitney U test for continuous variables and Fisher’s exact test for categorical variables, provided each group contained at least three counts. Differences in ANA antibody fluorescence patterns were evaluated using the Chi-square test. A significance level of 0.05 was set for all analyses. All statistical analyses were conducted using the JASP software version 0.17.2 (University of Amsterdam, Amsterdam, The Netherlands), which is based on the R programming language (https://jasp-stats.org/ [accessed on 12 September 2024]).

## 3. Results

### 3.1. Characteristics of Participants and Results of Autoantibody Testing

**Figure 1:**
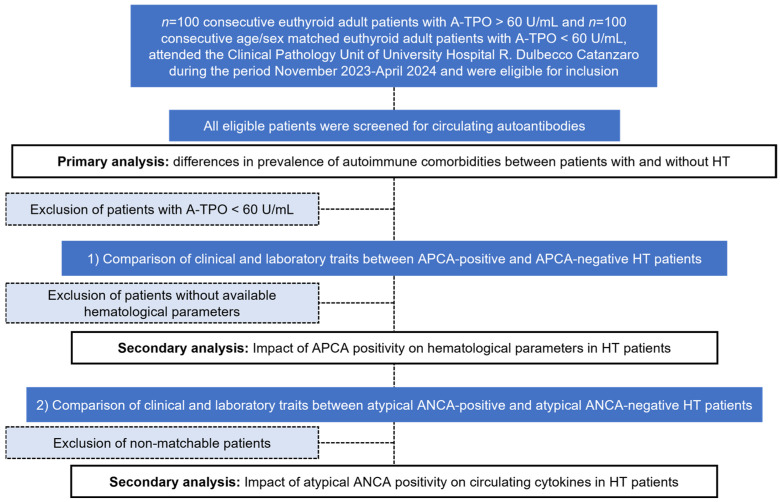
Flowchart of the study.

In this cross-sectional study, 200 euthyroid adult patients were consecutively enrolled over five months from the Clinical Pathology Unit at the University Hospital “R. Dulbecco” for thyroid function and HT screening. HT was diagnosed based on elevated A-TPO antibodies, indicating autoimmune thyroid involvement. A-TSH-R positivity was identified in six cases, with nearly all A-TPO-positive participants meeting the diagnostic criteria for HT. Using a 1:1 enrollment ratio, we selected 100 participants diagnosed with HT and 100 without, matched for age and sex to control for these variables. All participants underwent comprehensive serum screening for circulating autoantibodies, both organ-specific and non-organ-specific, to evaluate potential associations and comorbid conditions linked to thyroid autoimmunity. Figure 1 briefly illustrates the flowchart of the study.

Table 1 presents the demographics and results of autoantibody testing for the study population. Consistent with existing literature on the increased incidence of thyroid autoimmune diseases in women and older adults, 77.0% of the HT-positive participants were female, with a median age of 56 years. The control group, defined by their negative A-TPO status, had a similar age and sex distribution, confirming the adequacy of the matching procedure. Regarding autoantibody prevalence, none of the HT-negative individuals tested positive for A-Tg antibodies, in line with the inclusion criteria. In contrast, 60.0% of those diagnosed with HT based on A-TPO levels exhibited positive ATg antibodies, confirming overlapping autoantibody profiles in autoimmune thyroid disorders. However, the titers of A-Tg were significantly lower than those of anti-TPO, reinforcing the primary role of A-TPO antibodies in HT pathogenesis [11].

All participants met euthyroid criteria for inclusion. Nonetheless, HT-positive individuals had significantly higher average TSH levels compared to HT-negative individuals (median TSH: 2.215 vs. 1.705 μIU/mL, *p* = 0.025), suggesting a trend toward hypothyroidism due to thyroid autoimmunity. When evaluating comorbid autoimmune conditions, both groups displayed similar levels of ASCA and ASCG antibodies, indicating a comparable prevalence of Crohn’s disease-associated biomarkers across both cohorts [26]. ACCP antibody levels were predominantly negative in both groups, though this does not exclude the possibility of rheumatoid arthritis or other connective tissue diseases [27]. HT-positive participants exhibited a higher prevalence of positive ANCA antibodies (27.3% vs. 10.2%, *p* = 0.003), primarily showing an atypical fluorescence pattern, and ANA antibodies (68.4% vs. 44.9%, *p* = 0.001). This suggests a potential link between HT and systemic autoimmune conditions, although non-specific or false-positive results cannot be ruled out. No observed differences were found in the fluorescence patterns of ANA antibodies, with a speckled pattern being the most common in both groups nor in the proportion of ANA at high titers (>1:320), a marker often associated with SLE and other severe rheumatic diseases [28] (Table 1).

In examining specific autoimmune endocrine gland diseases associated with HT, we observed a nominal difference in the prevalence of positive anti-adrenal antibodies. These antibodies were more frequently found in patients with positive A-TPO antibodies compared to the control group (3 patients vs. none). However, the limited sample size may hinder the detection of rare endocrine conditions, such as Addison’s disease. In contrast, no difference was noted between the two groups regarding the presence of positive anti-islet antibodies, which are typically linked to incident type 1 diabetes and latent autoimmune diabetes in adults (LADA). Notably, when assessing the specific association with thyro-gastro autoimmunity, the prevalence of positive APCA antibodies was four times higher in HT patients compared to those with negative A-TPO antibodies (16.3% vs. 4.1%, *p* = 0.008) (Table 1).

### 3.2. Predictors of Thyro-Gastro Autoimmunity

The gut, stomach, and thyroid originate from the same embryological tissues, which may explain their functional and morphological similarities [29,30]. This shared developmental origin supports the associations among HT, celiac disease, and atrophic gastritis, particularly when immune tolerance is disrupted. Thyro-entero-gastro autoimmunity can lead to significant health issues such as anemia, micronutrient deficiencies, and malabsorption of medications, including hormone treatments for hypothyroidism, along with an increased risk of malignancies [31,32,33]. These conditions are often subtle, which can result in delayed diagnosis and treatment. While detection and public awareness of celiac disease have improved, reducing the number of undiagnosed cases and enhancing access to gluten-free diets, these advancements may inadvertently mask anti-tissue transglutaminase serology results, even in affected individuals [34]. This phenomenon is illustrated in Table 1, which shows no significant difference in anti-tissue transglutaminase antibodies between HT-positive and HT-negative groups. Conversely, atrophic gastritis often goes undetected, and APCA antibodies may serve as important biomarkers for this condition. In our study, we explored predictors of APCA positivity to identify which HT patients might benefit from proactive screening for atrophic gastritis. As detailed in Table 2, patients with positive APCA antibodies were significantly older (median age, 65.5 vs. 55.0 years, *p* = 0.002) and had higher titers of A-TPO antibodies (all above the upper limit of quantification) than those without APCA antibodies. These findings suggest a more severe disruption of thyro-gastro immunotolerance, highlighting the need for targeted screening strategies in this subgroup. Proactive screening for APCA positivity, particularly in older patients with active HT, could be advantageous. The nominal differences in mean corpuscular volume (MCV) and the significantly reduced red blood cell (RBC) count in HT patients with available hematological data, as shown in Table 3, suggest a trend towards macrocytic anemia in those presenting with APCA positivity. This further emphasizes the potential clinical benefits of such screening. In fact, 25% of APCA-positive patients (4 out of 16) were also positive for IFAB antibodies, which are associated with impaired vitamin B12 absorption and may contribute to the development of macrocytic anemia [35].

### 3.3. Significance of Atypical ANCA Positivity

In the search for predictors of atypical ANCA positivity among patients with HT, no statistically significant differences in clinical or serological characteristics were observed between patients with and without positive ANCA antibodies, as shown in Table 4. However, patients who tested positive for atypical ANCA antibodies were more likely to also test positive for APCA antibodies, suggesting a potential link with thyro-gastro autoimmunity.

**Figure 2:**
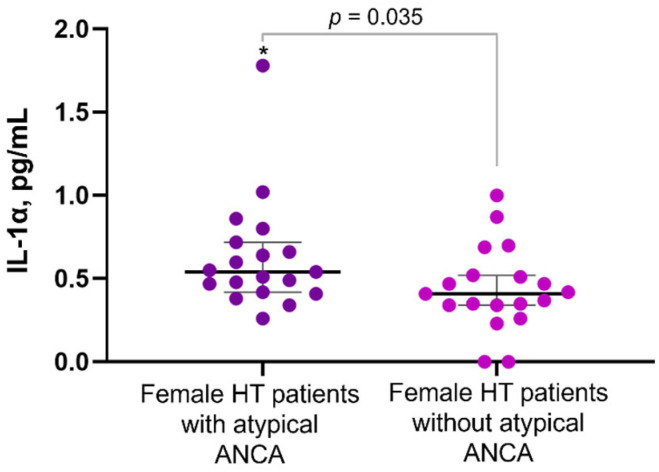
Differences in circulating IL-1α levels in female HT patients with and without atypical ANCA positivity. “*” denotes statistical significance.

Atypical ANCA, which targets multiple antigens, has been identified in various non-vasculitis conditions, such as ulcerative colitis and drug reactions [36]. Despite these associations, the clinical and laboratory significance of atypical ANCA in HT remains unclear. Given the high prevalence of atypical ANCA among patients with positive A-TPO antibodies (27.2%, as noted in Table 1), this is likely more than an incidental finding. We investigated whether their presence might indicate a more severe chronic pro-inflammatory state in HT patients. This elevated inflammatory state could significantly impact morbidity and mortality, underscoring the need for further research into the role of atypical ANCA in thyroid autoimmunity and gastrointestinal autoimmune disorders. In our analysis of serological inflammation markers in age- and sex-matched cohorts (26 individuals each), we found a pro-inflammatory cytokine profile in HT patients with atypical ANCA, including borderline significantly elevated median levels of IL-1α, a key driver of inflammation-related organ and tissue dysfunction [37]. We also observed a trend toward increased levels of IL-2, IL-4, and IL-8 (Table 5). Interestingly, when male patients were excluded from the analysis, the difference in circulating IL-1α levels reached statistical significance (median IL-1α: 0.54 vs. 0.41 pg/mL, *p* = 0.035), suggesting that atypical ANCA-associated inflammation in HT may be influenced by sex (Figure 2).

## 4. Discussion

Positivity to a variety of autoimmune markers frequently occurs in HT. Numerous studies have investigated this phenomenon across different populations, employing diverse selection criteria and analytical methods. Consequently, the reported co-occurrence of autoimmune diseases in HT varies significantly across the literature. In this study, we examined several serological markers of both organ-specific and systemic autoimmune diseases in a local population of adult, euthyroid, A-TPO-positive patients, comparing them to A-TPO-negative controls. Our primary findings confirm the significant association of HT with autoimmune gastritis (AIG), a relationship that has been well-documented in previous studies [33,38,39,40,41,42]. Additionally, we observed an association between A-TPO positivity and atypical ANCA, a connection that has received far less attention in the literature. The link between HT and AIG has been recognized for many years, initially as part of the “thyrogastric syndrome” and more recently categorized under “polyglandular autoimmune syndrome type IIIb”, where HT is a central disorder [19,29,43]. This connection is not surprising given that, despite their differing functions and lack of physical proximity, thyroid follicular cells and gastric parietal cells share common structural and biochemical features. These similarities likely arise from their shared embryological origin in the primitive foregut, and include the expression of Na+/I- symporter and similar peroxidase-active enzymes. Moreover, similar pathogenic mechanisms have been proposed for both autoimmune processes [29,30]. It is estimated that around 40% of patients with pernicious anemia, a condition that results from AIG, are also affected by HT [44,45]. Conversely, studies suggest a high prevalence (ranging from 12% to 40%) of AIG in HT [33,38,39,40,41,42], highlighting a bidirectional relationship between thyroid and gastric autoimmune disorders.

In this study, we found that more than 16% of HT patients were positive for APCA, with anti-TPO-positive patients having an approximately fourfold increased risk of AIG compared to controls. While these results align with previous findings, variations in ethnicity, age, recruitment criteria, and the methodologies used to measure APCA may explain the broad range of prevalence reported in the literature. Notably, most previous studies utilized qualitative or semiquantitative APCA assays based on indirect immunofluorescence (IIF), whereas our study employed a quantitative FEIA assay targeting H+/K+ ATPase. While there is no universally accepted gold standard for APCA testing, IIF, ELISA, and FEIA methods are all considered to have high sensitivity and specificity. However, IIF is known to be substrate- and operator-dependent, which can make it less standardizable than other techniques [46]. In line with previous works [39,47], our data show that age and A-TPO titer are associated with APCA. These findings may have important clinical implications, particularly in older patients with high A-TPO antibody levels. Specifically: (a) There is an increased susceptibility to AIG, which is clinically silent in most cases. AIG, being a precancerous lesion, may progress to gastric carcinoid tumor or even adenocarcinoma [31,32]. (b) Abnormal gastric pH can interfere with L-thyroxine absorption, potentially leading to inadequate hormonal replacement therapy in hypothyroid patients [33]. (c) Unexplained iron deficiency and/or pernicious anemia may also occur. These considerations highlight the need to screen for APCA in patients with HT, even in the absence of dyspeptic symptoms. Although this study did not find a significant impact on hematological parameters, it should be noted that similar results have been reported by others [48]. The progression from AIG to gastric atrophy, which may initially result in hypochlorhydic-dependent iron-deficiency anemia and later pernicious anemia, can take several years [49]. Therefore, the detection of APCA as a predictor of gastric mucosal atrophy may be valuable in adults with HT, even before hematological changes become apparent, for early diagnosis, monitoring, and treatment of the condition [42].

ANCA encompasses a group of autoantibodies initially screened using IIF. These antibodies are commonly used in the diagnosis of vasculitides—specifically, the typical ANCA patterns, which include cytoplasmic ANCA (cANCA) and perinuclear ANCA (pANCA). In addition to vasculitides, ANCA is also found in drug-induced vasculitis, inflammatory bowel disease (IBD), primary sclerosing cholangitis, and rheumatoid arthritis—conditions that are associated with atypical ANCA. The target antigens recognized by ANCA include proteins located in the cytoplasmic granules of neutrophils and in the lysosomes of monocytes. The most frequent of these are myeloperoxidase (MPO) and proteinase 3 (PR3), which are typically targeted by typical ANCA. Less common ANCA antigens include elastase, cathepsin G, anti-bacterial/permeability-increasing protein, lactoferrin, azurocidin, and lysozyme [50,51]. A peculiar finding in this study is the significant association between atypical ANCA and A-TPO, with 27% serological positivity to ANCA in A-TPO-positive patients compared to 10% in A-TPO-negative controls. In individuals with HT, the prevalence of systemic vasculitides and IBD is reported to be quite low [17]. Additionally, while thyroid disease is observed in about 20% of patients with ANCA-associated vasculitides [52] and hypothyroidism in 10% of cases [53], the association between ulcerative colitis and Hashimoto’s thyroiditis remains debated [43]. Based on these epidemiological and clinical data, the finding of nearly 30% ANCA positivity in A-TPO-positive patients requires further explanation. A previous Italian study demonstrated that approximately 18% of A-TPO-positive sera exhibit an ANCA pattern on ethanol-fixed neutrophils, and positivity persisted when using MPO-negative neutrophils as the substrate [54]. Furthermore, in patients with autoimmune thyroid disorders, ANCA positivity was detected in 28% of those with Graves’ Disease (GD) and 9% of those with HT, with only one GD patient showing MPO positivity [55]. In the same study, reactivity against lactoferrin was reported in 14% of GD cases and 18% of cases. Another study investigating ANCA positivity in patients with anti-microsomal antibodies confirmed the absence of cross-reactivity with MPO and identified elastase as the primary ANCA target [56]. The search for other potential ANCA target antigens is ongoing and may provide insight into the role of these autoantibodies in ANCA-associated diseases. As ANCA antigens are expressed on the surface of neutrophils once primed with specific antibodies and persist during apoptosis [50], atypical ANCA, however, appears unable to activate the oxidative burst of neutrophils in vitro, which suggests a limited pathogenetic role [57]. This aligns with the limited diagnostic utility of ANCA in IBD and related conditions, given their modest sensitivity and presence in both autoimmune and non-autoimmune disorders [58]. Although ongoing studies explore the clinical significance of atypical ANCA [59], we hypothesize that ANCA positivity may reflect the inflammatory milieu present in thyroid autoimmune diseases. A comparison of cytokine levels in HT patients, both with and without atypical ANCA, revealed significantly elevated IL-1α levels in female ANCA-positive patients, and a trend towards higher IL-1α levels in the broader HT population. To our knowledge, this is the first report of this association in the context of HT. Both IL-1α and IL-1β are key pro-inflammatory cytokines, although IL-1α’s role in autoimmune pathogenesis has only recently gained attention, likely because it is seldom detected in the circulation of patients with these conditions [37,60]. The increased sensitivity of our chemiluminescent immunoassay, which detects levels below the limits of traditional ELISA assays, may explain this finding [60]. Unlike IL-1β, which is not constitutively expressed and only produced during inflammation, IL-1α is present in a range of mesenchymal cells, particularly in barrier epithelia (e.g., gastrointestinal tract, lungs, skin) and vascular endothelium, as well as myeloid cells. When released in response to cell injury or death, IL-1α acts as an alarmin, triggering localized inflammation and tissue remodeling, which can contribute to organ dysfunction and amplify systemic immune responses [37]. These findings suggest that atypical ANCA positivity in HT patients may indicate a broader inflammatory status, a factor that endocrinologists should consider, especially in female patients, when evaluating potential comorbidities. Elevated IL-8, IL-10, IL-1β, and TNF-α levels have been reported in subclinical hypothyroid HT patients [61], though this study did not assess concurrent autoimmune diseases or the potential adverse metabolic impact of hypothyroidism. Our study also showed a high prevalence of ANA positivity. A report from China on hyperthyroid patients found higher ANA and ANCA positivity compared to controls (34% vs. 4% and 29% vs. 2%, respectively), though mostly with low titers [62]. Similarly, our findings in A-TPO-positive patients can be seen as a consequence of the inflammatory process associated with autoimmune disease [63].

Our study has some limitations, including a small sample size for intragroup comparisons and patient recruitment from a single center, which restricts the generalizability of our findings. Additionally, data on L-thyroxine replacement therapy to maintain euthyroidism, as well as information on dietary supplementation that may impact autoantibody levels, were not available. However, a key strength is the use of modern methodologies and consistent sources for all analyte measurements, ensuring analytical reliability.

## 5. Conclusions

This cross-sectional study confirms a significant association between HT and autoimmune gastritis, while also identifying a novel link between HT and atypical ANCA. Adult patients with HT exhibit a higher prevalence of circulating autoantibodies compared to controls, suggesting broader autoimmune dysfunction beyond the thyroid. These findings underscore the importance of comprehensive autoimmune screening in HT patients, especially in older individuals and those with elevated A-TPO levels. Early detection and management of comorbid autoimmune conditions could improve patient outcomes. Further studies with larger cohorts are needed to validate these results and explore the mechanistic connections between HT, atypical ANCA positivity, and systemic inflammation.

## Figures and Tables

**Table 1:** Characteristics of the study population.

	Non-HT Patients (A-TPO Negative, *n* = 100)	HT Patients (A-TPO Positive, *n* = 100)	*p* Value
Female, *n*	76 (76.0%)	77 (77.0%)	1.000
Age, y	55.0 (46.0–62.5)	56.0 (47.5–63.5)	0.689
A-TPO, U/mL	28.0 (28.0–33.4)	1300 (245–1300)	**<0.001**
A-Tg, U/mL	1.3 (1.3–1.3)	8.3 (2.2–48.1)	**<0.001**
A-Tg > 4.5 U/mL	0 (0.0%)	60 (60.6%) *	**<0.001**
TSH, μIU/mL	1.705 (1.120–2.660)	2.215 (1.135–4.155)	**0.025**
ASCA, U/mL	2.1 (1.2–3.9)	2.2 (1.0–4.1)	0.790
ASCG, U/mL	1.3 (0.7–2.9)	1.7 (0.6–4.4)	0.302
ACCP, U/mL	1.1 (0.9–1.3)	1.0 (0.8–1.2)	0.254
tTG IgA, U/mL	0.5 (0.3–0.8)	0.5 (0.3–0.7)	0.785
tTG IgG, U/mL	0.8 (0.6–0.9)	0.7 (0.6–0.8)	0.109
APCA > 1:80, *n*	4 (4.1%) *	16 (16.3%) *	**0.008**
AMA > 1:80, *n*	3 (3.0%) *	0 (0.0%)	n.a.
GAD > 10 U/mL, *n*	3 (3.0%)	2 (2.0%)	n.a.
IA2 > 10 U/mL, *n*	1 (1.0%)	3 (3.0%)	n.a.
ZNT8 > 15 U/mL, *n*	2 (2.0%)	0 (0.0%)	n.a.
ANCA > 1:20, *n*	10 (10.2%)	27 (27.3%) *	**0.003**
ANA > 1:80, *n*	44 (44.9%) *	67 (68.4%) *	**0.001**
ANA > 1:320, *n*	18 (18.2%) *	22 (22.5%) *	0.596
Fluorescence ANA pattern:			0.322 **
- Homogeneous	5 (11.6%)	4 (6.0%)	
- Speckled	28 (65.1%)	37 (55.2%)	
- Nucleolar	6 (14.0%)	14 (20.9%)	
- Mixed/Other	4 (9.3%)	12 (17.9%)	
Screen ratio	0.2 (0.1–0.3)	0.1 (0.1–0.2)	0.259
ADA > 1:10, *n*	0 (0.0%)	3 (3.0%)	n.a.

* valid percentage, calculated by excluding missing individual patient data from the analysis; ** calculated using the Chi-squared test. A-TPO, anti-thyroperoxidase antibodies; A-Tg, anti-thyroglobuline antibodies; TSH, thyrotropin stimulating hormone; ASCA, anti-saccharomyces cerevisiae antibodies IgA; ASCG, anti-saccharomyces cerevisiae antibodies IgG; ACCP, anti-cyclic citrullinated peptides antibodies; tTG IgA, anti-transglutaminase antibodies IgA; tTG IgG, anti-transglutaminase antibodies IgG; APCA, anti-parietal cells antibodies; AMA, anti-mitochondrial antibodies; GAD: glutamic acid decarboxylase; IA2, anti-tyrosin phosphatase A2 antibodies; ZnT8, anti-zinc transporters 8 antibodies; ANCA, anti-neutrophil cytoplasmic antibodies; ANA, antinuclear antibodies; ADA, anti-adrenal cortex antibodies. Screen Ratio: ratio is automatically calculated by the instrument by comparing the signal of the test sample with that of the calibrators, specifically for U1RNP (RNP70, A, C), SS-A/Ro (60 kDa, 52 kDa), SS-B/La, Centromere B, Scl-70, Jo-1, Fibrillarin, RNA Pol III, Rib-P, PM-Scl, PCNA, Mi-2, Sm and DNA. Bold values denote statistical significance at the *p* < 0.05 level. n.a.: not applicable due to insufficient count numbers.

**Table 2:** Comparison between APCA-negative and APCA-positive patients with HT.

	HT Patients APCA-Negative (*n* = 82) *	HT Patients APCA-Positive (*n* = 16)	*p* Value
Female, *n*	61 (74.4%)	14 (87.5%)	0.345
Age, y	55.0 (46.0–61.0)	65.5 (57.7–72.0)	0.002
A-TPO, U/mL	919 (232–1300)	1300 (1300–1300)	0.020
A-Tg, U/mL	8.8 (1.9–48.9)	7.4 (2.6–51.6)	0.957
A-Tg > 4.5 U/mL	50 (61.7%)	9 (56.3%)	0.781
TSH, μIU/mL	2.495 (1.215–4.413)	2.030 (0.900–2.967)	0.263
ASCA, U/mL	2.1 (0.9–4.5)	2.7 (1.6–3.5)	0.735
ASCG, U/mL	1.7 (0.6–4.6)	1.1 (0.7–3.9)	0.567
ACCP, U/mL	1.0 (0.8–1.2)	1.1 (1.0–1.3)	0.177
GAD > 10 U/mL, *n*	1 (1.2%)	0 (0.0%)	n.a.
IA2 > 10 U/mL, *n*	2 (2.4%)	1 (6.3%)	n.a.
ZNT8 > 15 U/mL, *n*	0 (0.0%)	0 (0.0%)	n.a.
ANCA > 1:20, *n*	20 (24.4%)	7 (43.8%)	0.132
ANA > 1:80, *n*	57 (71.2%)	9 (56.3%)	0.252
Screen ratio	0.2 (0.1–0.2)	0.1 (0.1–0.2)	0.811
ADA > 1:10, *n*	2 (2.4%)	1 (6.3%)	n.a.

* two patients were excluded from the main HT cohort due to unavailable APCA screening test results because of insufficient aliquot sample. Bold values denote statistical significance at the *p* < 0.05 level. n.a.: not applicable due to insufficient count numbers.

**Table 3:** Impact of APCA positivity on hematological parameters in patients with HT.

	HT Patients APCA-Negative (*n* = 25) *	HT Patients APCA-Positive (*n* = 10) *	*p* Value
Hb, g/dL	13.3 (12.8–14.4)	13.0 (12.1–13.4)	0.242
MCV, fl	88.3 (82.6–90.0)	91.1 (86.7–92.9)	0.121
RBC, *n* × 10^6^/μL	4.8 (4.6–5.2)	4.5 (1.1–4.7)	0.047
WBC, *n* × 10^3^/μL	7.2 (5.3–9.0)	6.8 (5.7–7.4)	0.969
RDW	13.5 (12.9–13.8)	13.2 (13.0–13.7)	0.913

* HT patients without available hematological parameters were excluded from this secondary analysis. Hb, hemoglobin; MCV, mean corpuscular volume; RBC, red blood cells; WBC, white blood cells; RDW, red blood cells distribution width. The bold value denotes statistical significance at the *p* < 0.05 level.

**Table 4:** Comparison between atypical ANCA negative and atypical ANCA positive patients with HT in relation to serological test results.

	HT Patients Atypical ANCA-Negative (*n* = 72) *	HT Patients Atypical ANCA-Positive (*n* = 27)	*p* Value
Female, *n*	56 (77.8%)	20 (74.1%)	0.790
Age, y	55.0 (46.0–62.0)	60.0 (50.0–68.5)	0.111
A-TPO, U/mL	1300 (241–1300)	1300 (423–1300)	0.542
A-Tg, U/mL	7.3 (2.2–44.0)	14.9 (1.9–93.1)	0.699
A-Tg > 4.5 U/mL	41 (57.8%)	18 (66.7%)	0.493
TSH, μIU/mL	1.995 (0.912–3.790)	2.960 (1.859–4.375)	0.114
ASCA, U/mL	2.0 (1.0–4.3)	2.8 (0.9–4.0)	0.917
ASCG, U/mL	1.4 (0.6–4.1)	3.0 (0.7–7.2)	0.285
ACCP, U/mL	1.0 (0.8–1.3)	1.0 (0.9–1.2)	0.751
GAD > 10 U/mL, *n*	2 (2.8%)	0 (0.0%)	n.a.
IA2 > 10 U/mL, *n*	1 (1.4%)	2 (7.4%)	n.a.
ZNT8 > 15 U/mL, *n*	0 (0.0%)	0 (0.0%)	n.a.
APCA > 1:80, *n*	9 (12.7%)	7 (25.9%)	0.132
ANA > 1:80, *n*	49 (69.0%)	17 (65.4%)	0.807
Screen ratio	0.1 (0.1–0.2)	0.2 (0.1–0.3)	0.243
ADA > 1:10, *n*	2 (2.8%)	1 (3.7%)	n.a.

* one patient was excluded from the main HT cohort due to the unavailability of ANCA screening test results due to insufficient aliquot sample. n.a.: not applicable due to insufficient count numbers.

**Table 5:** Comparison between atypical ANCA-negative and atypical ANCA-positive patients with HT in relation to circulating cytokines.

	HT Patients Atypical ANCA-Negative (*n* = 26)	HT Patients Atypical ANCA-Positive (*n* = 26 *)	*p* Value
IL-2, pg/mL	0.0 (0.0–0.0)	0.0 (0.0–2.5)	0.819
IL-4, pg/mL	0.0 (0.0–2.2)	0.7 (0.0–1.5)	0.960
IL-6, pg/mL	1.3 (0.8–2.1)	1.6 (0.8–2.6)	0.595
IL-8, pg/mL	11.5 (7.6–22.6)	13.7 (6.5–45.2)	0.985
IL-10, pg/mL	0.0 (0.0–1.4)	0.0 (0.0–1.3)	0.921
VEGF, pg/mL	175.1 (107.5–290.4)	170.6 (113.0–224.6)	0.927
INFγ, pg/mL	0.0 (0.0–0.0)	0.0 (0.0–0.0)	0.667
TNFα, pg/mL	2.6 (2.1–3.8)	2.8 (2.5–3.4)	0.812
IL-1α, pg/mL	0.4 (0.3–0.5)	0.5 (0.4–0.7)	0.068
IL-1β, pg/mL	0.0 (0.0–1.8)	0.0 (0.0–1.4)	0.215
MCP1, pg/mL	332.8 (241.6–429.6)	303.0 (262.6–422.1)	0.855
EGF, pg/mL	111.6 (61.2–161.6)	104.2 (67.1–150.1)	0.935

* one patient with atypical ANCA was excluded from the main HT cohort due to insufficient aliquot sample.

## Data Availability

Data supporting the reported results are available from the corresponding author upon reasonable request.
